# Previously Unreported Chromosomal Aberrations of t(3;3)(q29;q23), t(4;11)(q21;q23), and t(11;18)(q10;q10) in a Patient with Accelerated Phase Ph+ CML

**DOI:** 10.1155/2014/582016

**Published:** 2014-02-23

**Authors:** Cigdem Aydin, Zafer Cetin, Ozan Salim, Orhan Kemal Yucel, Levent Undar, Sibel Berker Karauzum

**Affiliations:** ^1^Department of Medical Biology and Genetics, School of Medicine, Akdeniz University, 07058 Antalya, Turkey; ^2^Department of Hematology, School of Medicine, Akdeniz University, 07058 Antalya, Turkey

## Abstract

Chronic myelogenous leukemia (CML) is a clonal hematological disorder, which is characterized by the presence of the classical or variant Philadelphia translocations. During the progression to blastic phase of the disease secondary chromosomal abnormalities may emerge. Such secondary chromosomal abnormalities are nonrandom, the more frequent ones being trisomy 8 and 19, supernumerary i(17q), and extra Philadelphia chromosomes. Furthermore, a minor percentage of the patients may acquire different secondary chromosomal abnormalities including translocations between other chromosomes. We report here a patient with Ph+ CML presenting secondary chromosomal abnormalities including t(4;11)(q21;q23), t(3;3)(q29;q23) and t(11;18)(q10;q10) during the course of CML progression.

## 1. Introduction

Chronic myelogenous leukemia (CML) is a clonal myeloproliferative disorder of primitive hematopoietic stem cells, which is characterized by the presence of the classical or variant Philadelphia translocations. The appearance of secondary chromosomal abnormalities in CML patients usually is considered as the hallmark of the blastic phase. Around 80% of patients in blast crisis show chromosome abnormalities in addition to the Ph chromosome, and 75% of these show at least one of +8, double Philadelphia chromosome, or i(17q) [1]. However, a minor percentage of the patients show different secondary chromosomal abnormalities. We report here a patient with Ph+ CML presenting secondary chromosomal abnormalities including t(4;11)(q21;q23), t(3;3)(q29;q23), and t(11;18)(q10;q10).

## 2. Case Report

**Figure 1:**
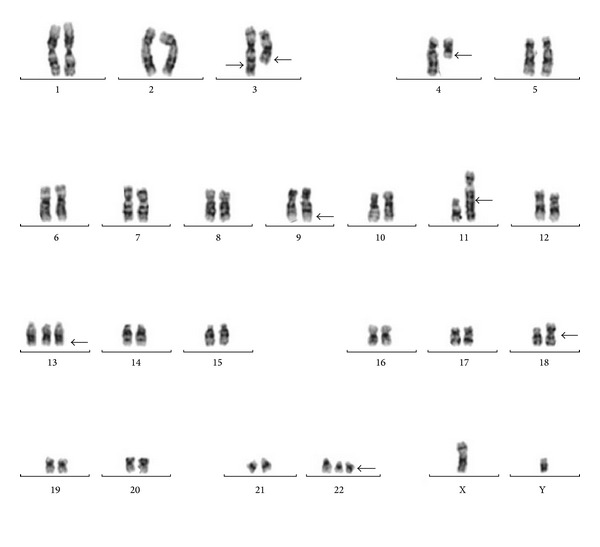
Karyotype of the bone marrow cells evaluated in 2012.

**Figure 2:**
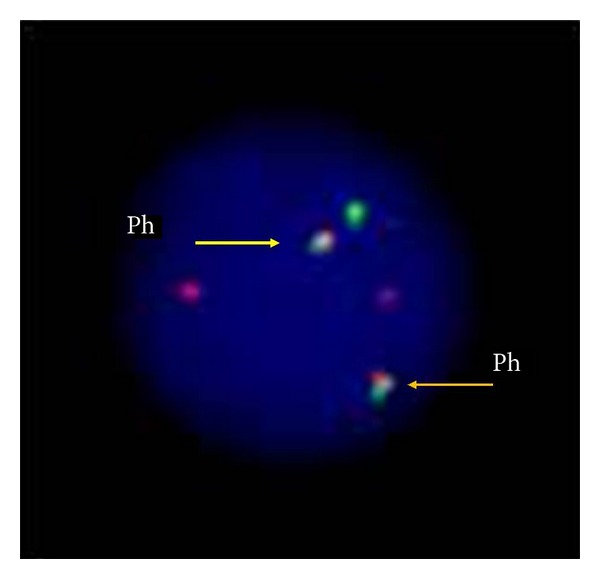
An interphase cell having a secondary Philadelphia chromosome detected in 2012.

In July 2002, a 55-year-old male patient presented with abdominal discomfort, splenomegaly, and leukocytosis (120 × 10^9^ cells/L). Bone marrow aspiration and biopsy bone marrow were consistent with chronic myelogenous leukemia (CML) with less than 5% blasts, and he was diagnosed with chronic phase CML. Hydroxyurea followed by interferon alfa plus low dose cytarabine was given between 2002 and 2004. In 2004, specific fusion gene BCR-ABL was detected in peripheral blood sample by real-time polymerase chain reaction assay. Treatment was switched to imatinib mesylate (400 mg/day), when available in Turkey. Although hematological remission was achieved at the third month of therapy, cytogenetic and molecular remissions could not be reached at the end of the first year of therapy. Follow-up cytogenetic analysis in 2008 revealed the following complex chromosomal abnormalities: 46, XY, t(4;11)(q21;q23), t(9;22)(q34;q11), and t(11;18)(q10;q10)[13]. The patient was accepted as accelerated phase CML according to cytogenetic evidence of clonal evaluation. Subsequently, the dose of imatinib mesylate was raised to 600 mg daily. The patient was lost to follow-up between 2009 and 2011. In 2011, same complex karyotype was observed in bone marrow sample at repeated analyse and dasatinib was started 100 mg daily. But it had to be ceased due to recurrent symptomatic grade 4 cytopenias in a couple of months after the dasatinib use. Conventional cytogenetic analysis was repeated in 2012. The karyotype was as follows: 46, XY, t(3;3)(q29;q23), t(4;11)(q21;q23), t(9;22)(q34;q11), t(11;18)(q10;q10)[80]/48, XY, t(3;3)(q29;q23), t(4;11)(q21;q23), t(9;22)(q34;q11), t(11;18)(q10;q10), +13, der(22) t(9;22)(q34;q11)[20] (Figure 1). Trisomy 13, t(3;3), and secondary Philadelphia chromosome were detected in addition to t(4;11), t(9;22), and t(11;18) translocations. FISH analysis showed that 61% and 30% of the cells had one and two Philadelphia chromosomes, respectively (Figure 2). In 2012, he was still in accelerated phase and nilotinib was initiated 800 mg daily. However, it was discontinued due to grade 4 cytopenias within three months. Finally, he was admitted to inpatient ward because of blastic crisis. Last karyotype analysis was performed at this time. All secondary abnormalities were observed in 25% of bone marrow cells but extra Philadelphia chromosome was seen only in one metaphase. t(9;22) and double Philadelphia chromosomes were detected in 81% and 8% of the analyzed interphase cells with FISH analysis, respectively. He refused to receive further multiagent chemotherapy and was lost to follow-up.

## 3. Discussion

CML is a clonal hematological disorder, which progresses from a chronic phase to more aggressive phases named accelerated phase and blastic crisis [2]. Additional chromosomal abnormalities are found in 10–20% during chronic phase and in 60–80% preceding or accompanying blastic crisis. There is a strong association between secondary changes and blastic transformation. After the detection of these secondary chromosomal abnormalities disease may progress to blastic crisis by weeks or months [1]. Four major changes occur in more than 70% of patients: trisomies of the chromosomes 8 and 19, i(17q), and extra Philadelphia chromosomes [2]. Other less common chromosomal abnormalities such as trisomy 19, trisomy 21, trisomy 17, and monosomy 17 can also be observed during blastic transformation [3]. In CML cases with acquired complex karyotype, it has been suggested that sequence of the secondary chromosomal aberrations is random and generally associated with the previously administered treatment agent [3], and also, mechanisms leading to this genetic instability are not exactly resolved. In our case, we detected a complex karyotype including t(3;3)(q29;q23), t(4;11)(q21;q23), and t(11;18)(q10;q10) translocations after treatments with interferon alfa and imatinib mesylate.

After the treatment with dasatinib, trisomy 13 and a secondary Philadelphia chromosome were emerged in addition to chromosomal abnormalities mentioned above. Translocation t(4;11)(q21;q23) leading to formation of MLL/AF4 fusion gene has been observed in nearly 10% of newly diagnosed adult patients with B-cell acute lymphoblastic leukemia [4]. Although our case does not have the typical biological, immunophenotypic, and clinical features of the cases with MLL/AF4 fusion gene we applied FISH analysis by using MLL break apart probe, indicating that MLL gene was intact in our case. This result shows that t(4;11) translocation observed in our case was not classical t(4;11)(q21;q23) translocation leading to MLL/AF4 fusion gene. To our knowledge, t(3;3)(q29;q23) and t(11;18)(q10;q10) translocations are also not reported previously in a case with accelerated or chronic phase CML. t(4;11)(q21;q23) and t(11;18)(q10;q10) translocations may suggest that these translocations resulted in the formation of leukomogenic fusion genes leading to transformation to accelerated phase in our case. Five months after the first detection of the clones including t(3;3)(q29;q23), +13, and secondary Philadelphia chromosome in addition to t(4;11)(q21;q23) and t(11;18)(q10;q10) translocations, the patient has been following up as blastic phase CML.

To our knowledge these chromosomal abnormalities are unique in a case with Philadelphia positive CML. Our patient was in accelerated phase CML at the time of detection of these secondary chromosomal abnormalities but they precede the blastic crisis as expected.
